# Characterization of Antioxidant Constituents and Geographical Differentiation of *Polygonum multiflorum* Thunb. Leaves Using 2D-LC-ECD and UHPLC-Orbitrap-MS/MS

**DOI:** 10.3390/antiox15081008

**Published:** 2026-08-13

**Authors:** Zhengyan Tan, Pei Xu, Dan Wang, Zongna Han, Huaying Chen, Qianqian Zhu, Yang Yang, Wei Pan, Piao Wang, Rongxiang Chen

**Affiliations:** 1 School of Basic Medicine, Zunyi Medical University, Zunyi 563000, China; tanzhengyan@zmu.edu.cn (Z.T.); xupei@zmu.edu.cn (P.X.); 2 School of Pharmacy, Zunyi Medical University, Zunyi 563000, China; wangdan202607@163.com (D.W.); hanzongna29@163.com (Z.H.); huayingchen2026@163.com (H.C.); qianqianzhu0708@163.com (Q.Z.); yangyang08042026@163.com (Y.Y.); panwei@zmu.edu.cn (W.P.); wangpiao@zmu.edu.cn (P.W.)

**Keywords:** *Polygonum multiflorum* Thunb. leaves, antioxidant activity, 2D-LC-ECD, LC-MS/MS, statistical analysis, quantitative determination

## Abstract

This study established an activity-oriented analysis method based on offline two-dimensional liquid chromatography–electrochemical detection (2D-LC-ECD) combined with ultra-high-performance liquid chromatography–Orbitrap-MS/MS (UHPLC-Orbitrap-MS/MS), which was used to comprehensively characterize the antioxidant components of *Polygonum multiflorum* Thunb. leaves (PML) and screen for differential compounds in samples from different origins. Antioxidant components were screened using 2D-LC-ECD combined with in vitro radical scavenging experiments; structure identification was conducted via UHPLC-Orbitrap-MS/MS, and chemometric analysis alongside quantitative determination of 19 major components was carried out across 31 batches of samples. A total of 43 potential antioxidant compounds were identified from PML, including 11 phenolic acids, 29 flavonoids, 2 stilbene glycosides, and 1 amino acid; phenolic acids and their derivatives, along with flavonoids, were identified as major contributors to antioxidant activity. Hierarchical clustering analysis and principal component analysis both grouped samples into four categories by origin; the orthogonal partial least squares discriminant analysis model (R^2^X = 0.933, R^2^Y = 0.941, Q^2^ = 0.846) screened 10 key markers (VIP > 1). The quantitative results showed that polygonacetophenoside had the highest content in PML. In summary, the 2D-LC-ECD coupled with LC-Orbitrap-MS/MS strategy confirmed that phenolic acids and flavonoids are the core material basis for PML’s antioxidant capacity, providing scientific evidence for the industrial development of PML as a natural antioxidant.

## 1. Introduction

*Polygonum multiflorum* Thunb. (*P. multiflorum*) is a traditional tonic Chinese medicinal herb belonging to the Polygonaceae family [1]. In modern pharmacological studies, the root of *P. multiflorum* has been demonstrated to possess antioxidant, neuroprotective, and anti-hair loss effects [2,3,4,5]. Guizhou is the primary production area for *P. multiflorum*. Modern pharmacological studies have confirmed that the tuberous root of *P. multiflorum* possesses various pharmacological activities, including antioxidant, anti-aging, lipid-regulating, and anti-atherosclerotic effects [6,7]. Among these, its excellent antioxidant capacity serves as the core material basis for its multifaceted health-promoting and therapeutic effects. Chemical composition studies indicate that the tuberous root primarily contains active components such as diphenylstyryl glycosides, anthraquinones [8], phenolic acids, and flavonoids [9]. Among these, 2,3,5,4′-tetrahydroxydiphenylstyryl-2-O-β-D-glucoside has the highest content and exhibits the most significant activity [10].

Unlike the well-studied root tuber, *P. multiflorum leaves* (PML) have long been neglected in terms of their medicinal potential. Systematic investigations on PML remain limited, and the corresponding chemical composition and pharmacological mechanisms warrant further elucidation. In regions such as Jiangxi and Taiwan, there is a folk tradition of drying the leaves and using them as a herbal tea [11]. Modern pharmacological research has confirmed that PML extract possesses multiple bioactivities, including antioxidant, anti-inflammatory, lipid-regulating, hypoglycemic, and neuroprotective effects [11,12]. Its mechanisms involve scavenging free radicals, inhibiting α-glucosidase and acetylcholinesterase, and regulating the NF-κB pathway [13]. Unlike that of the root tuber, the chemical composition of PML is dominated by flavonoids, phenolics, and polysaccharides, with extremely low levels of stilbene glycosides and anthraquinones [14,15,16]. The unique composition and developmental potential make PML a natural antioxidant resource with significant application prospects. However, systematic analysis and fundamental research on the active antioxidant components in PML remain relatively limited.

The chemical constituents of natural plants are complex and diverse. Due to limited peak capacity, conventional one-dimensional liquid chromatography (1D-LC) is prone to severe peak co-elution during separation [17,18]. Two-dimensional liquid chromatography (2D-LC), by employing an orthogonal separation mode, can significantly increase peak capacity and resolution, thereby enabling the efficient separation and analysis of complex samples [19,20,21]. The electrochemical detector detects compounds by measuring the current generated from redox reactions occurring on the electrode [22]. During the oxidation or reduction of electroactive substances, the current generated at the working electrode is directly proportional to the concentration of the analyte [23]. Therefore, electrochemical detection (ECD), with its high sensitivity and specificity, has become a powerful tool for the targeted screening of antioxidants. Given ECD’s excellent selectivity for redox-active compounds such as phenols and flavonoids, this technique offers significant advantages in the evaluation of antioxidant activity [24]. Its further coupling with UHPLC-Orbitrap-MS/MS enables rapid screening and identification of antioxidant components. Previous studies have successfully employed offline 2D-LC-ECD coupled with LC-MS/MS to screen and identify antioxidant active constituents from the leaves of various plants, validating the feasibility of this strategy [25,26].

The antioxidant efficacy of natural plants results from the synergistic action of multiple components. Additionally, the geographical origins and ecological conditions of Chinese herbal medicines significantly regulate the synthesis of plant secondary metabolites [27], directly influencing the chemical composition, content, and pharmacological quality of the herbs, and serving as the core factors driving quality variations in Chinese herbal medicines.

In the present study, offline 2D-LC-ECD, UHPLC-Orbitrap-MS/MS and in vitro radical scavenging assays were integrated to rapidly screen and identify antioxidants in PML. LC-HRMS profiling of multiple batches of samples, combined with chemometric models including hierarchical cluster analysis (HCA), principal component analysis (PCA), and orthogonal partial least squares discriminant analysis (OPLS-DA), was performed to explore crucial differential compounds associated with antioxidant discrepancies among geographical populations. Quantitative analysis of the major antioxidant-related components was also performed to elucidate the molecular basis of the antioxidant activity in PML, thereby providing a scientific basis for its quality control and further development and utilization.

## 2. Materials and Methods

### 2.1. Materials

Quercitrin, rutin, isoquercitrin, chlorogenic acid, epicatechin, catechin, nicotiflorin, trifolin, isoschaftoside, schaftoside, isovitexin, and hyperoside were purchased from Pufei De Biotechnology Co., Ltd. (Chengdu, Sichuan, China). Gallocatechin, caffeic acid, tryptophan, gallic acid, epicatechin gallate, quercetin-7-glucoside, and methyl gallate were obtained from Aladdin Biochemical Technology Co., Ltd. (Shanghai, China). LC-MS grade formic acid (>98%), HPLC grade methanol (>98%), and LC-MS grade acetonitrile (>99.9%) were purchased from Aladdin Biochemical Technology Co., Ltd.; all reference standards had a purity of ≥98%, while the remaining reagents were analytical grade. The water used in the experiment was ultrapure water prepared by an Elix Advantage ultrapure water system (Merck Millipore, Billerica, MA, USA).

A total of 31 fresh PML samples were collected from major traditional production areas within Guizhou Province. The plant material was authenticated by Dr. Meng Lingjie from Zunyi Medical University (Zunyi, China). Detailed sample information is listed in Table 1. Samples were dried at 40 °C, pulverized using a BJ-800A high-speed pulverizer (Baijie, Shanghai, China), passed through a 60-mesh sieve, and then stored at 4 °C.

### 2.2. Preparation of PML Extract

Approximately 1.0 g of PML powder was accurately weighed and extracted with 5 mL of 80% (*v*/*v*) methanol aqueous solution under ultrasonication (40 kHz, 60 min) for 2D-LC separation. For UHPLC-Q-Orbitrap-HRMS analysis, 1.0 g of the powder was extracted with 20 mL of the identical solvent system under the same ultrasonication conditions. Each extract was centrifuged at 9000 rpm for 5 min using a Microfuge-20 high-speed centrifuge (Thermo Fisher Scientific, Waltham, MA, USA), and the supernatant was filtered through a 0.22 μm filter membrane. The extract prepared for UHPLC-Q-Orbitrap-HRMS was further diluted 10-fold prior to injection.

For 2D-LC-ECD, a high concentration is necessary because the collected fractions undergo further dilution during the first-dimension separation. For UHPLC-Orbitrap-HRMS analysis, a considerably more dilute extract is used to avoid signal saturation and minimize matrix effects.

### 2.3. Preparation of the Reference Standard Solutions

An appropriate amount of each reference standard was accurately weighed and dissolved in methanol to prepare individual stock solutions (1 mg/mL), which were stored at −20 °C. Immediately before use, the stock solutions were mixed and diluted with 80% methanol to the required concentrations to prepare the mixed standard working solutions.

### 2.4. 2D-LC Separation of PML

#### 2.4.1. Conditions for the First-Dimension Liquid Chromatography

A 2695 HPLC system (Waters, Milford, MA, USA) equipped with a diode array detector (DAD) was used for the first-dimension liquid chromatography (^1^D-LC). The stationary phase was an RD-Phenyl-Hexyl column (10 mm × 150 mm, 5 µm, Zhongpu, Fujian, China). The mobile phase consisted of methanol (A) and an aqueous solution of 0.1% formic acid (B). The elution gradient was as follows: 0–25 min, 20–30% A; 25–50 min, 30–50% A; 50–60 min, 50–99% A; 60–65 min, 99% A; 65–67 min, 99–20% A; 67–75 min, 20% A. Chromatographic separation was performed under the following conditions: flow rate of 2.5 mL/min, injection volume of 100 µL, column temperature of 35 °C, and detection at 280 nm. During the 3.0–52.5 min gradient elution, 100 fractions were collected at 0.5 min intervals (each 1.25 mL). All fractions were stored at −20 °C until analysis, after which aliquots were taken for the second-dimension liquid chromatography (^2^D-LC) and antioxidant activity evaluation.

#### 2.4.2. Second-Dimension Liquid Chromatography Conditions

For the ^2^D-LC, an Ultimate 3000 bio-RS high-performance liquid chromatograph (Thermo Fisher Scientific, Waltham, MA, USA) was used with an ACQUITY HSS T3 (2.1 mm × 100 mm, 1.8 μm, Waters, Milford, MA, USA) as the stationary phase, with acetonitrile (A) and a 50 mmol/L citrate buffer (citric acid–potassium citrate buffer, pH = 2.59) (B) as the mobile phase. The detailed gradient elution program is shown in Table 2. The detection potential was set to 600 mV, the flow rate to 0.4 mL/min, the column temperature to 45 °C, the autosampler temperature to 15 °C, and the injection volume to 5 µL.

#### 2.4.3. UHPLC-Orbitrap-MS/MS Conditions for Fraction Identification

The fractions collected from the ^1^D-LC were analyzed using a Vanquish-F UHPLC system (Thermo Fisher Scientific, Waltham, MA, USA) under the following conditions. An ACQUITY HSS T3 column (2.1 mm × 100 mm, 1.8 µm, Milford, MA, USA) was used as the stationary phase, with acetonitrile (A) and an aqueous solution of 0.1% formic acid (B) as the mobile phase. The specific elution gradient is the same as that in ^2^D-LC, as shown in Table 2. The flow rate was 0.3 mL/min, the injection volume was 10 µL, the column temperature was 45 °C, and the sample temperature was 12 °C.

Mass spectrometry was performed using an Orbitrap Exploris 120 high-resolution mass spectrometer (Thermo Fisher Scientific, Waltham, MA, USA) under the following conditions: the ion source was heated electrospray ionization (HESI) in negative ion mode; the spray voltage was 2.5 kV; sheath gas flow was 35 Arb; auxiliary gas flow was 10 Arb; evaporator temperature was 350 °C; and ion transfer tube temperature was 320 °C. The scanning mode was full scan MS/dd-MS^2^. The full scan range was *m*/*z* 150–1500, with a resolution of 60,000. MS/MS resolution was 15,000, and high-energy collision-induced dissociation was set to stepwise mode (20%, 40%, and 60%). Data acquisition and processing were performed using TraceFinder 4.1.

### 2.5. Antioxidant Activity Evaluation of the ^1^D-LC Fractions

#### 2.5.1. DPPH Radical Scavenging Capacity Assay

The 1,1-diphenyl-2-picrylhydrazyl (DPPH) radical scavenging assay was employed, with slight modifications to the established method [28]. A 200 µL aliquot of ^1^D-LC fraction of PML (fractions collected at 3.0, 3.5, 9.0, 9.5, 10.0, 24.0, and 24.5 min were further diluted 5-fold with 80% methanol) was taken, and 100 µL of DPPH (0.1 mmol/L in methanol) reagent was added. The mixture was incubated in the dark at room temperature for 30 min before the absorbance was measured at 517 nm using a microplate reader (Thermo Fisher Scientific, Waltham, MA, USA). Eighty percent methanol was used as the blank, and Trolox served as the positive control. A standard curve was plotted, the DPPH radical scavenging capacity was calculated as Trolox equivalents, and the results were expressed as μg of Trolox equivalents per mL of fraction (µg TE/mL).

#### 2.5.2. ABTS Radical Scavenging Capacity Assay

The 2,2′-azobis-3-ethylbenzothiazoline-6-sulfonic acid diammonium salt (ABTS) radical scavenging assay was employed, with slight modifications to the established method [29]. Briefly, 200 µL of PML fractions (with fractions collected at 3.0, 3.5, 9.0, 9.5, 10.0, 24.0, and 24.5 min further diluted 5-fold with 80% methanol) were mixed with 100 µL ABTS working solution, whose absorbance was adjusted to 0.70 ± 0.1 at 734 nm. After reacting in the dark for 30 min, the absorbance was measured at 734 nm. The blank and positive control settings were identical to those of the DPPH assay. The ABTS scavenging activity was quantified as Trolox equivalents, and the results were expressed as µg of Trolox equivalents per mL of fraction (µg TE/mL).

### 2.6. Analysis and Quantitative Determination of Major Antioxidant Components in PML from Different Regions

#### 2.6.1. UHPLC-Orbitrap-MS/MS Analytical Conditions

The Vanquish-F UHPLC-Orbitrap Exploris 120 high-resolution mass spectrometer was used to analyze 31 batches of PML extracts. The stationary phase was XBridge BEH C18 (2.1 × 150 mm, 2.5 μm, Waters, Milford, MA, USA), and the mobile phase was acetonitrile (A) and 0.1% formic acid (B). The elution gradient was as follows: 0–18 min, 95–50% B; 18–20 min, 50–30% B; 20–21 min, 30–2% B; 21–25 min, 2% B; 25–27 min, 2–95% B; 27–34 min, 95% B. The flow rate was 0.3 mL/min, the column temperature was 45 °C, the sample temperature was 15 °C, and the injection volume was 1 µL. Mass spectrometry conditions were the same as those used for mass spectrometry in ^2^D-LC fraction identification.

#### 2.6.2. Method Validation

Intra-day precision was evaluated by six consecutive injections of sample S1 within one day, and inter-day precision was determined by continuous detection for three days. Relative standard deviation (RSD) of peak areas was calculated. Linear regression was established by plotting the peak area on the y-axis against the concentration on the x-axis, using mixed standard solutions at a series of concentrations. The LOD and LOQ were determined based on signal-to-noise ratios (S/N), where the LOD and LOQ correspond to S/N ≥ 3 and S/N ≥ 10, respectively, using serial dilutions of the mixed standard solutions. The recovery test was performed by adding standard compounds to leaf samples with known component contents. Six parallel samples were prepared and analyzed to calculate average recovery. Matrix effects were assessed by comparing the slopes of solvent calibration curves and PML matrix-matched calibration curves.

### 2.7. Data Processing and Statistical Analysis

Compound Discoverer 3.4 and SIRIUS CSI:FingerID 6.3 [30] were applied for peak picking and compound identification via database matching. The peak areas of the 43 identified antioxidants were extracted using Compound Discoverer 3.4. The resulting data matrix was normalized by the total peak area of each sample to correct for minor injection volume deviations and instrumental response drift, followed by Pareto scaling prior to multivariate analysis. Multivariate statistical analysis including HCA and PCA was performed using Origin 2021, while OPLS-DA was conducted using SIMCA 14.1 software.

## 3. Results

### 3.1. Separation and Identification of Antioxidant Compounds in PML Using 2D-LC-ECD

#### 3.1.1. 2D-LC-ECD Separation of PML

**Figure 1:**
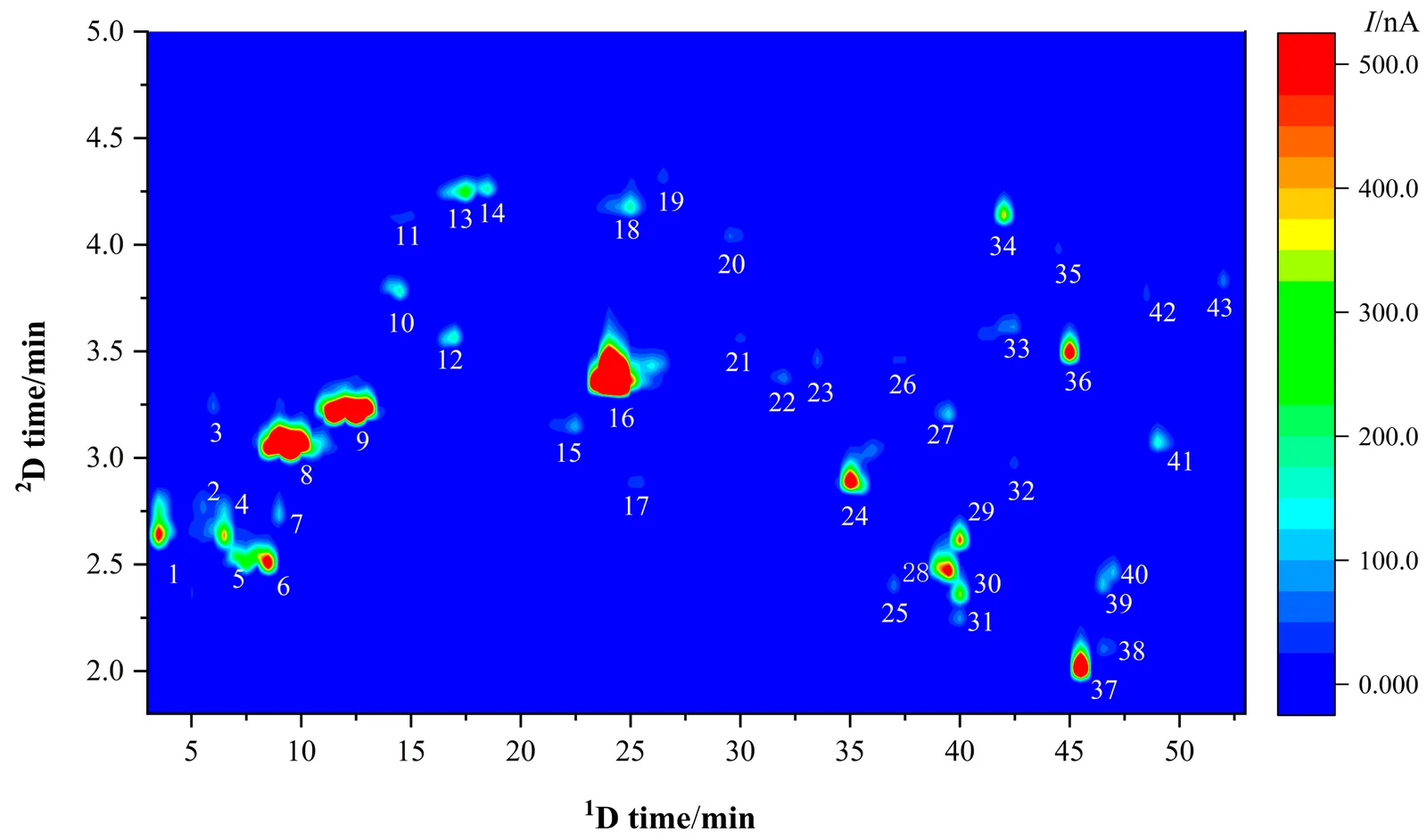
2D-LC-ECD chromatogram of PML.

Compared with 1D-LC, 2D-LC significantly improves both peak resolution and separation efficiency. In this study, an offline reversed-phase × reversed-phase (RP × RP) two-dimensional mode was employed. In the first dimension, a Phenyl-Hexyl column was used as the stationary phase, and methanol-0.1% formic acid aqueous solution was used as the mobile phase for gradient elution, with fractions collected in segments. In the second dimension, since ECD typically requires a higher buffer salt concentration to ensure baseline stability [31], an acetonitrile-citrate solution was selected as the mobile phase and an HSS T3 column as the stationary phase. The stationary and mobile phases of the two-dimensional system exhibit different selectivities, ensuring orthogonality of separation. Furthermore, this mode has been successfully applied to the separation of flavonoids and phenolic compounds in medicinal and edible plants such as *Zanthoxylum bungeanum* leaves and licorice [32,33]. The 2D-LC-ECD chromatogram of PML is shown in Figure 1. The peaks with higher responses are labeled 1–43, visually indicating the chemical components in PML that exhibit strong reducing properties on the ECD electrode surface. The 43 major peaks are evenly distributed, representing a significant improvement in resolution compared to simple 1D-LC.

#### 3.1.2. Antioxidant Activity Evaluation of First-Dimension Fractions

**Figure 2:**
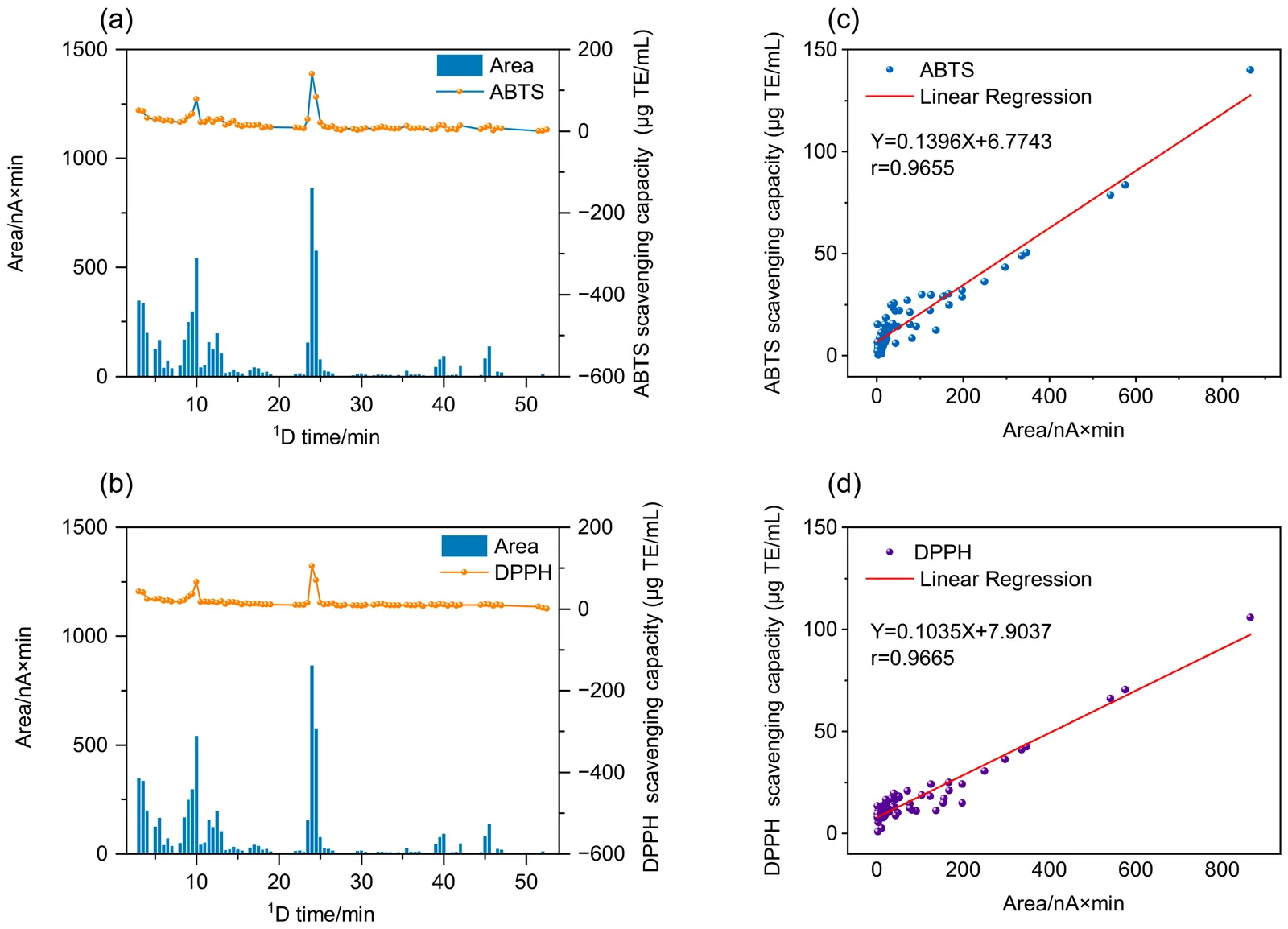
Peak areas and antioxidant activities of different first-dimension fractions. (**a**) ABTS radical scavenging capacity; (**b**) DPPH radical scavenging capacity; (**c**) linear correlation between peak areas and their ABTS radical scavenging capacity of different fractions; (**d**) linear correlation between peak areas and their DPPH radical scavenging capacity of different fractions.

To validate the use of LC-ECD as a reliable tool for antioxidant screening, the radical scavenging activities of the first-dimension fractions were assessed via ABTS and DPPH assays. The obtained data, calculated as radical scavenging capacity (µg TE/mL) to enable direct quantitative comparison across fractions, are depicted in Figure 2.

The results indicated that the fractions collected at different elution times exhibited varying degrees of antioxidant activity. Among them, fractions collected within the first 30 min demonstrated significantly stronger free radical scavenging capacity than those collected between 30 and 52 min. It is worth noting that fractions with larger peak areas, such as those collected at 3.0, 3.5, 9.0, 9.5, 10.0, 24.0, and 24.5 min, exhibited stronger radical scavenging capacity. These active fractions corresponded exactly to Peak 1, Peak 8, and Peak 16 in Figure 1, suggesting that these components may be the major antioxidant compounds in PML. The above results indicate that the major antioxidant components in PML are concentrated in the hydrophilic fractions; this finding provides a theoretical basis for the efficient extraction of natural water-soluble antioxidants.

Fraction-based antioxidant assessment is more convenient, rapid and efficient than traditional single-component separation and activity verification. Conventional methods are limited by low constituent content and unstable separation performance, restricting research on antioxidant bioactivity. Figure 2c,d demonstrate a significant positive correlation between the peak areas of 2D-LC-ECD fractions and their corresponding radical scavenging equivalents, with correlation coefficients of 0.9559 for ABTS assay and 0.9336 for DPPH assay. The favorable response of ECD towards antioxidants ensured a strong correlation between electrochemical signals and in vitro antioxidant capacity, validating the offline 2D-LC-ECD combined with radical scavenging assay as a precise screening approach for leaf antioxidants [34]. The ECD response originates from oxidation of electroactive groups; for phenolic antioxidants, it primarily depends on the number and activity of phenolic hydroxyl groups. In this study, the key antioxidants in PML—phenolic acids, flavonoids, and stilbenes—are all phenolic derivatives with similar molecular weight, polarity, and electroactive groups, ensuring a relatively uniform ECD response. Accordingly, peak areas correlated strongly with antioxidant capacity, further validating offline 2D-LC-ECD coupled with radical scavenging assays as a reliable screening approach. However, for plant systems with greater structural diversity—such as those containing thiols, heterocycles, or alkaloids—the ECD response may vary markedly with compound structure, potentially weakening the peak area-antioxidant capacity correlation. Therefore, using ECD peak area as a quantitative antioxidant indicator requires evaluation of the structural features of antioxidants in the specific sample.

#### 3.1.3. UHPLC-Orbitrap-MS/MS Identification of the Fractions

As shown in Figure 1, multiple chromatographic peaks with distinct electrochemical responses were observed in the PML extract, suggesting a potential abundance of chemically active components with antioxidant properties.

To further identify the chemical components corresponding to each response peak, this study selected the time interval from 3 to 52.5 min, where electrochemical responses were relatively concentrated, and conducted UHPLC-Orbitrap-MS/MS analysis. Using Compound Discoverer combined with SIRIUS CSI:FingerID, the main chemical components corresponding to the 43 peaks were identified. The names, molecular formulas, and MS/MS data of each compound are shown in Table 3. Based on the structural characteristics of the parent compounds, the 43 identified compounds were classified into four major categories: phenolic acids and their derivatives, primarily centered on gallic acid and caffeic acid derivatives; flavonoids, mainly including flavanols and their derivatives, such as quercetin, apigenin, kaempferol, and their derivatives; stilbene glycosides, which feature a stilbene parent nucleus and are characteristic components of PML; and amino acids. The chromatographic peaks with the highest responses in Figure 1 were caffeic acid (6), catechin (8), chlorogenic acid (9), methyl 4,6-di-O-galloyl-β-D-glucopyranoside (24), hyperoside (28), kaempferol glucoside (36), and quercetin (37). All compounds were initially identified by fragment analysis using LC-HRMS; except for peaks 24 and 36, the rest of the substances were further confirmed by comparison with standard samples. The above compounds are the main antioxidant components of PML.

Among them, there are 11 phenolic acids and their derivatives, which are further classified into three subclasses based on parent nucleus structures and substituent types. Specifically, gallic acid and its derivatives represent the dominant subclass, encompassing 8 compounds in total: gallic acid, galloyl-glucoside, methyl 4,6-di-O-galloyl-β-D-glucopyranoside, polygoacetophenoside, polygoacetophenoside-glucoside, two isomers of polygoacetophenoside-glucoside, and feruloyl-homogalloyl-glucose. These compounds contain the galloyl structural unit and represent a class of phenolic acid structures known for their strong free radical scavenging capacity. There are three compounds in the caffeic acid group, namely caffeic acid, caffeoylglucoside, and chlorogenic acid. In addition, another phenolic acid derivative, ferulic acid glucoside, was identified.

Flavonoids were the most abundant class of compounds identified in this study, comprising a total of 29 compounds and accounting for the majority of all identified compounds. Based on differences in their parent ring structures, the flavanols comprised 9 compounds, including gallocatechin, catechin, epicatechin, epicatechin gallate, gallocatechin-O-β-D-glucuronide, procyanidin B, epigallocatechin-epicatechin, epigallocatechin-epicatechin isomer, and procyanidin B dimer monoglucoside. Notably, the fractions containing catechin exhibited significant free radical scavenging activity, indicating that the flavonoid antioxidant potential in PML also merits attention. Quercetin and its derivatives included seven compounds: hyperoside, isoquercitrin, rutin, quercitrin, quercetin-7-glucoside, quercetin-3-O-neohesperidoside, and quercetin-3-O-(6″-O-caffeoyl)-β-D-glucoside. Apigenin and its derivatives totalled eight compounds, including apigenin-O-glucoside, isovitexin, carlinoside, schaftoside, isoschaftoside, caffeoyl apigenin-7,4′-di-O-β-D-glucoside, and caffeoyl apigenin-O-glucose-O-arabinoside. Kaempferol and its derivatives consisted of three compounds, namely trifolin, kaempferol glucoside, and kaempferol diglucoside. One structurally unique polymethoxyflavonol was classified as other flavonoids: 5,7,3′,6′-tetrahydroxy-8,2′-dimethoxyflavone 6′-O-glucoside. Two stilbene glycosides were identified: pentahydroxystilbene diglucoside and an acylated stilbene glycoside. This class represents a characteristic group of compounds that distinguishes PML from other plant sources; while they are widely present in the roots and vines of *P. multiflorum*, fewer compounds of this class have been identified in the leaves. One amino acid was identified: tryptophan.

The antioxidant activity of PML is primarily attributed to phenolic acids and flavonoids. Phenolic acids such as gallic acid and caffeic acid derivatives, and galloylated procyanidins scavenge free radicals via hydrogen atom transfer and single electron transfer mechanisms, with multiple galloyl groups providing abundant phenolic hydroxyls that stabilize phenoxyl radicals and enhance reducing capacity [35]. Flavonoids, particularly flavan-3-ols such as catechin, epicatechin, and gallocatechin, and flavonol glycosides such as hyperoside, isoquercitrin, quercitrin, and rutin, exert antioxidant effects through the catechol moiety in the B-ring and the 3-OH substituent in the C-ring, which are essential for metal chelation and DPPH/ABTS radical scavenging [36]. These findings are consistent with previous pharmacological reports demonstrating that PML extracts modulate oxidative stress via NF-κB pathway regulation and free radical neutralization [12], and that the tuberous root’s antioxidant and anti-aging effects are linked to similar polyphenolic scaffolds [6,10]. Thus, the phenolic acids and flavonoids identified in the present study constitute the core material basis for the documented antioxidant activity of PML.

Currently, analytical methods for antioxidant components in medicinal and edible plants such as PML primarily rely on HPLC coupled with UV or LC-MS. These methods are widely used due to their high sensitivity and ability to identify a wide range of compounds [37,38,39]. However, the separation of complex mixtures with similar polarity or low concentrations, as well as certain unstable antioxidants and those present in trace amounts, remains a significant challenge. 2D-LC effectively addresses these shortcomings by significantly improving separation efficiency and peak capacity, making it particularly suitable for the separation of complex matrices such as plant extracts [40,41]. Targeted antioxidant screening via ECD greatly accelerates the discovery of bioactive components and lays a foundation for leaf quality evaluation.

### 3.2. Comparison of Antioxidant Components in PML from Different Regions

**Figure 3:**
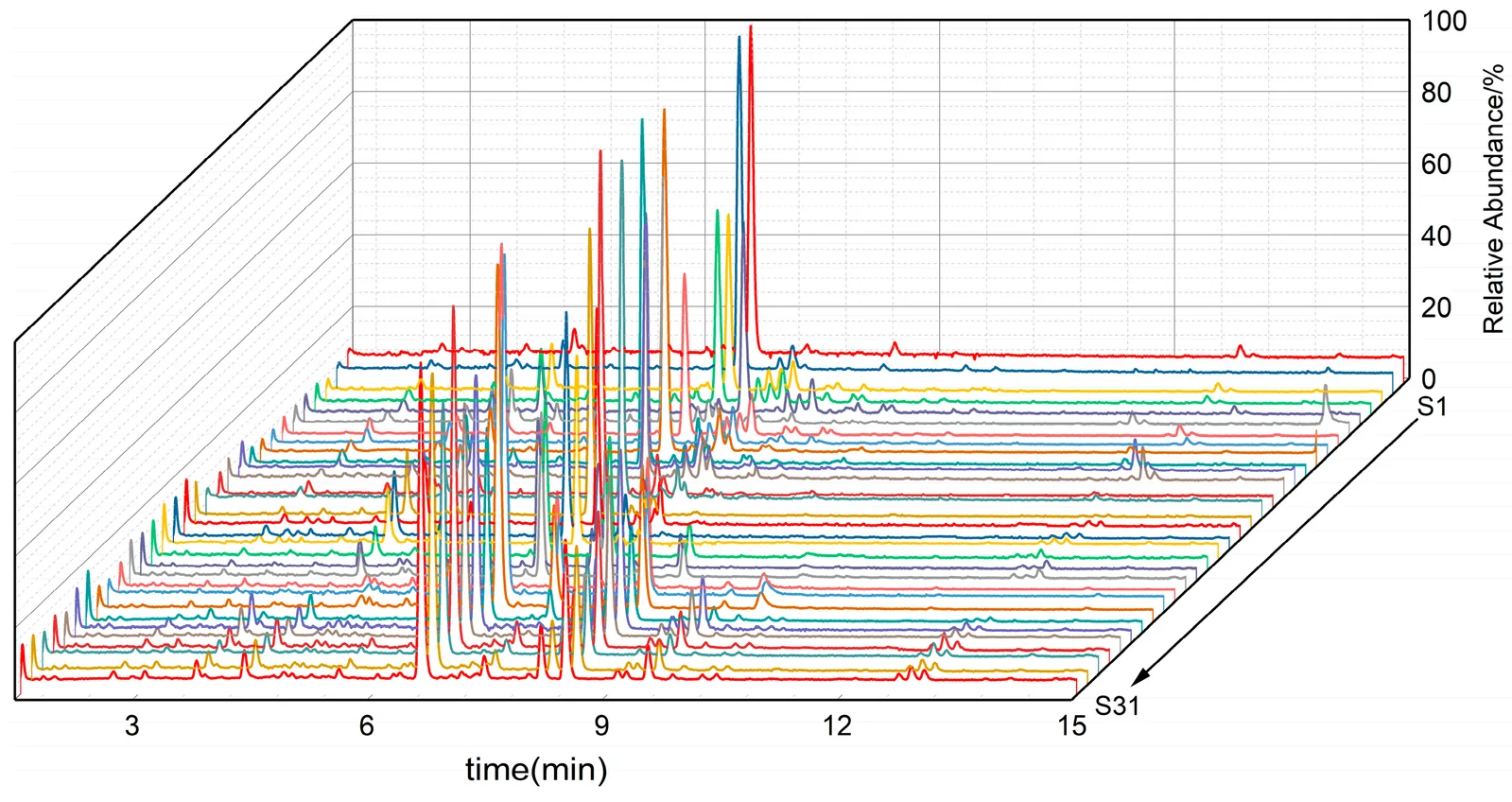
Total ion current chromatogram of the quality profile for 31 batches of PML.

To compare their antioxidant profiles, 31 batches of PML samples collected from different regions in Guizhou Province were analyzed by LC-HRMS with a focus on the 43 previously identified antioxidant compounds. The total ion chromatograms of the samples are shown in Figure 3. The peak areas of the 43 identified antioxidants were extracted using Compound Discoverer 3.4 for multivariate statistical analysis including HCA, PCA, and OPLS-DA.

#### 3.2.1. HCA

**Figure 4:**
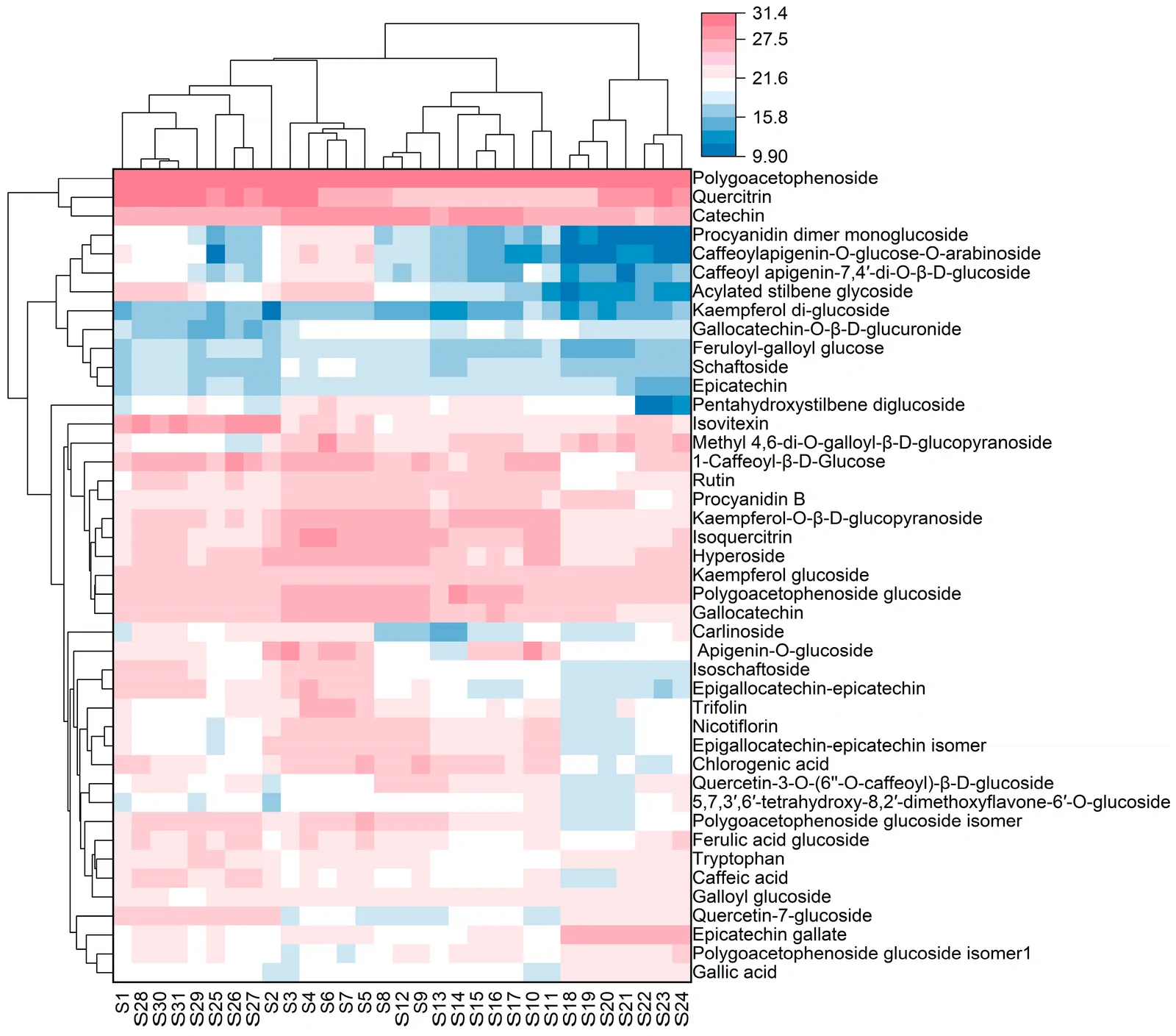
Heat map of cluster analysis results for 31 batches of PML samples.

To systematically evaluate the overall chemical similarities and differences in PML from different geographical origins, hierarchical cluster analysis based on Euclidean distance was performed on the standardized peak areas of 43 main antioxidant compounds from 31 batches of samples. The clustering algorithm used was the between-groups average linkage method, and the results are shown in Figure 4.

The clustering dendrogram clearly shows that the 31 batches of PML samples exhibit a highly origin-specific clustering pattern. At a Euclidean distance of 30, they cluster into one category, indicating similarity among samples. All samples are clearly divided into four independent clusters, with each cluster corresponding exactly to a single geographical origin; no cross-clustering of samples across origins was observed. Systematic cluster analysis based on the 43 compounds enables effective differentiation of the 31 batches of PML samples. This classification more precisely reflects the chemotaxonomic relationships among samples, with samples within the same group exhibiting higher homogeneity in overall characteristics. Furthermore, this study found significant differences in intra-group homogeneity among PML samples from different regions, with the Congjiang group showing the highest internal homogeneity, while the Qinglong group exhibited relatively greater internal variation. This phenomenon may be related to the microenvironmental heterogeneity of the sampling sites. The clustering results perfectly matched the collection regions, providing a chemotaxonomic basis for subsequent group validation and the screening of characteristic indicators.

#### 3.2.2. PCA

**Figure 5:**
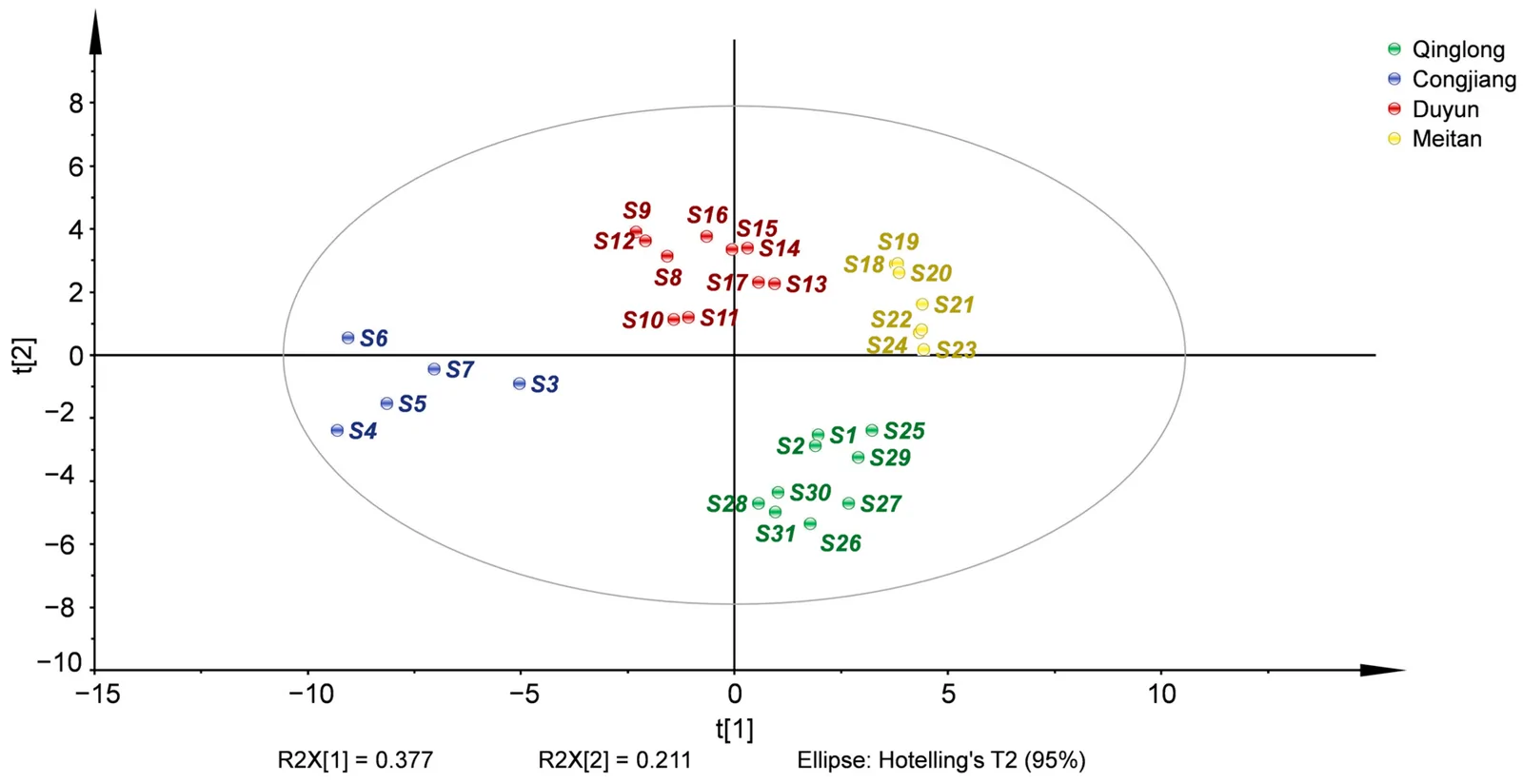
PCA scores plot of the 31 batches of PML samples (R2X = 0.824, Q2 = 0.610).

PCA is the most widely used unsupervised multivariate statistical dimensionality reduction method in natural product research [42,43,44,45]. It can reduce high-dimensional variables to a few core principal components while retaining as much of the original data information as possible, objectively reflecting the natural grouping patterns of samples and overall intergroup differences, and avoiding the subjective grouping bias of supervised models. Therefore, this study also performed the unsupervised principal component analysis, with the results shown in Figure 5. The analysis revealed that the first principal component explains 37.7% of the total variance in the original data (R2X[1]), while the second principal component explains 21.1% (R2X[2]), with the cumulative explanatory power of the first two principal components reaching 58.8%. In multivariate statistical analysis of complex natural product systems, this cumulative explanation rate adequately represents the overall characteristic information of the samples, indicating good model fit and reliable analytical results [46]. All 31 samples fell completely within the 95% confidence ellipse without any outliers, indicating good data quality, reproducibility, and stability in this analysis, with no abnormal data interfering with the results.

Regarding the spatial distribution, samples from the four origins exhibited clear classification patterns with tight intra-group clustering and significant inter-group separation. This intuitively revealed the overall differences in chemical composition among samples from different origins. Specifically, samples from the Congjiang origin were exclusively concentrated along the negative axis of PC1, achieving complete spatial separation from the other three origins. This indicates that the Congjiang group possesses the most distinct chemical composition. Samples from the Duyun origin were entirely concentrated in the lower half of the positive PC2 axis and near the origin of PC1, achieving complete vertical separation from the Qinglong samples along the PC2 axis and forming a significant horizontal distinction from both the Congjiang and Qinglong origin samples along the PC1 axis. Samples from the Meitan origin were primarily clustered in the intermediate region between the positive axis of PC1 and the origin, exhibiting the highest intra-group clustering. This indicates that samples from this origin demonstrate the best chemical composition homogeneity and stability. Samples from Qinglong were distributed along the positive PC2 axis, showing a clear separation trend from the Duyun samples on the PC2 axis; the overall characteristics of these two groups were relatively close but still effectively distinguishable. The PCA results clearly confirm that there are significant regional differences in the overall chemical composition of PML from the four origins (Qinglong, Congjiang, Duyun, and Meitan), and that origin is the key factor influencing the accumulation of their chemical components.

Furthermore, these natural clustering results are fully consistent with the classification results of hierarchical cluster analysis based on Euclidean distance. The mutual corroboration of these two unsupervised multivariate statistical methods further enhances the reliability and robustness of the analysis results, laying a solid foundation for subsequent supervised pattern recognition analysis, the screening of region-specific marker compounds, and the establishment of a quality evaluation system.

#### 3.2.3. OPLS-DA

**Figure 6:**
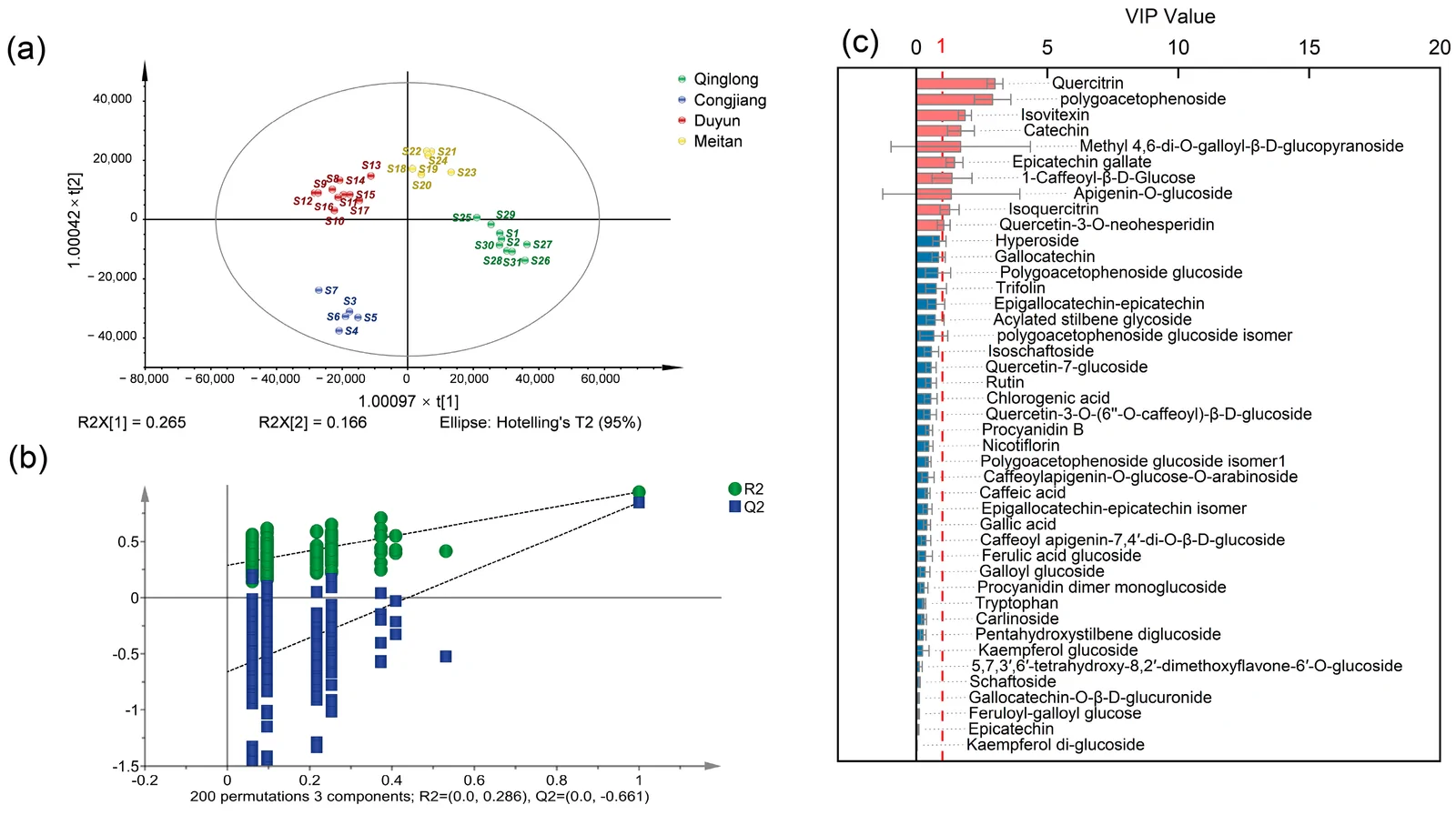
Results of the OPLS-DA model analysis for 31 batches of PML samples: (**a**) OPLS-DA score plot with R^2^X = 0.933, R^2^Y = 0.941, Q^2^ = 0.846; (**b**) Results from 200 random permutations [R^2^ = (0.0, 0.286); Q^2^ = (0.0, −0.661)]; (**c**) VIP values of OPLS-DA. Note: “×” indicates the multiplication sign.

To identify the marker compounds responsible for the differences in the chemical composition of PML samples from different origins, this study used the peak areas of the 43 compounds as independent variables and sample origin as the classification dependent variable. An OPLS-DA model was constructed using SIMCA software, and the results are shown in Figure 6. The model parameters R^2^X, R^2^Y, and Q^2^ describe the goodness of fit and predictive ability of the model, respectively. An R^2^X value closer to 1 indicates a more stable model, while a higher R^2^Y reflects stronger explanatory power. All samples fell within the 95% confidence ellipse, and a Q^2^ value greater than 0.5 indicates good predictive ability.

The model fit and predictive performance parameters show that the cumulative explanatory power of the OPLS-DA model for the independent variable matrix is R^2^X = 0.933, and for the dependent variable is R^2^Y = 0.941. The model’s predictive performance parameter obtained from cross-validation is Q^2^ = 0.846. All three core parameters are greater than 0.5, indicating that the model possesses excellent explanatory power for sample classification, good fit, and stable and reliable predictive capability. The OPLS-DA scatter plot Figure 6a shows that PML samples from the four origins (Qinglong, Congjiang, Duyun, and Meitan) exhibit significant trends of tight intra-group clustering and clear inter-group separation. All samples are distributed within the 95% confidence ellipse without any outlier samples, indicating good stability of the chemical profiles of samples from different origins and that the geographic origin is the core driving factor for chemical differences in PML.

To validate the robustness of the model and rule out the risk of overfitting, a permutation test with 200 permutations was performed on the dependent variable (sample origin) (Figure 6b). The intercept values of R^2^ = (0.0, 0.286) and Q^2^ = (0.0, −0.661) confirmed that the model was not overfitted. All randomly permuted Q^2^ values were negative and substantially lower than the original Q^2^ (0.846). A Q^2^ > 0.5 combined with a negative Q^2^ intercept is widely accepted as evidence of a valid and predictive OPLS-DA model. This confirms that the OPLS-DA model exhibits no overfitting, possesses strong statistical reliability, and can be used for the subsequent screening of differential marker compounds in PML from different origins.

To identify the components that contribute most significantly to the classification of PML, a VIP analysis was performed on the OPLS-DA model (Figure 6c), where VIP > 1 indicates that the component makes a significant contribution to the model.

Ten differential markers with VIP values above 1.0 were identified, namely quercitrin, polygoacetophenoside, catechin, isovitexin, methyl 4,6-di-O-galloyl-β-D-glucopyranoside, epicatechin gallate, apigenin-O-glucoside, isoquercitrin, 1-caffeoyl-β-D-glucose, and quercetin-3-O-neohesperidin. These compounds can serve as potential chemical indicators for antioxidant quality assessment and geographical traceability of PML.

Unlike previous studies that primarily relied on conventional 1D-LC-UV or 1D-LC-MS for profiling phenolic compounds in PML [15,16,47], the present study integrates offline 2D-LC-ECD with UHPLC-Orbitrap-MS/MS. This analytical strategy exhibits multiple remarkable advantages. The application of ECD enables activity-guided, highly sensitive screening of electrochemically active antioxidants. The RP × RP 2D-LC system effectively overcomes the co-elution bottleneck of structurally analogous flavonoids and phenolic acids by substantially enhancing peak capacity and separation resolution. Furthermore, the synergistic integration of high-resolution tandem mass spectrometric analysis and in vitro free radical scavenging activity validation further ensures the comprehensiveness and reliability of active compound identification.

Accordingly, the present study developed a hyphenated analytical platform coupling 2D-LC, ECD, and UHPLC-Orbitrap-MS/MS, which was employed for the elucidation of the antioxidant profile and geographical origin traceability of PML.

### 3.3. Quantitative Analysis of Major Antioxidant Components in PML from Different Regions

#### 3.3.1. Method Validation

The established UHPLC-Orbitrap-MS/MS quantification method was systematically validated in terms of linearity, LOD, LOQ, precision and recovery (Table A1). All 19 target compounds exhibited good linear relationships within their respective linear ranges, with R^2^ values all greater than 0.9900. The LOD and LOQ ranged from 0.01 to 0.57 μg/mL and 0.03 to 1.9 μg/mL, respectively. The RSD for intra-day precision ranged from 1.41% to 2.23%, and that for inter-day precision ranged from 0.87% to 1.45%, with both values below 5%. This indicates that the method exhibits good repeatability and reliable long-term stability.

The average spike recoveries ranged from 93.04% to 105.29%, with associated RSDs of 1.56–12.33%. The matrix effect values for all 19 target compounds fell within 92.05–109.98%, well inside the acceptable range of 80–120%, indicating minimal matrix interference. This finding demonstrates that solvent dilution can effectively mitigate matrix effects in complex plant samples. Overall, the validation parameters met the standard requirements for the simultaneous quantification of multiple constituents in natural medicinal plants, confirming the satisfactory accuracy of the established UHPLC-Orbitrap-MS/MS method.

#### 3.3.2. Quantification of 19 Components in 31 Batches of PML

**Figure 7:**
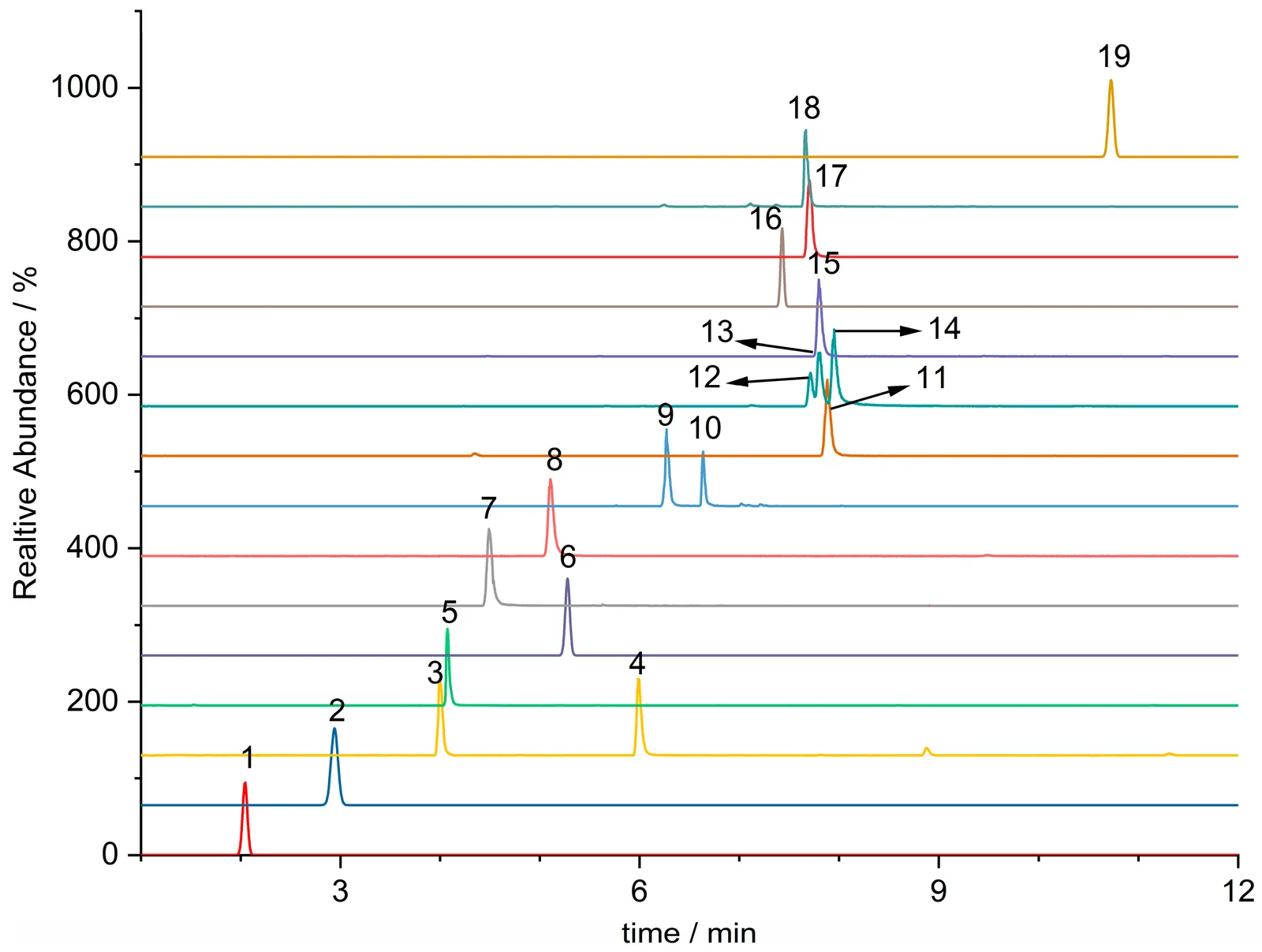
Standard reference solutions for 19 compounds in PML. Note: 1. Gallic acid, 2. Gallocatechin, 3. Catechin, 4. Epicatechin, 5. Tryptophan, 6. Caffeic acid, 7. Chlorogenic acid, 8. Polygoacetophenoside, 9. Isoschaftoside, 10. Schaftoside, 11. Epicatechin gallate, 12. Hyperoside, 13. Isoquercitrin, 14. Quercetin-7-glucoside, 15. Isovitexin, 16. Rutin, 17. Quercitrin, 18. Nicotiflorin, 19. Trifolin.

**Figure 8:**
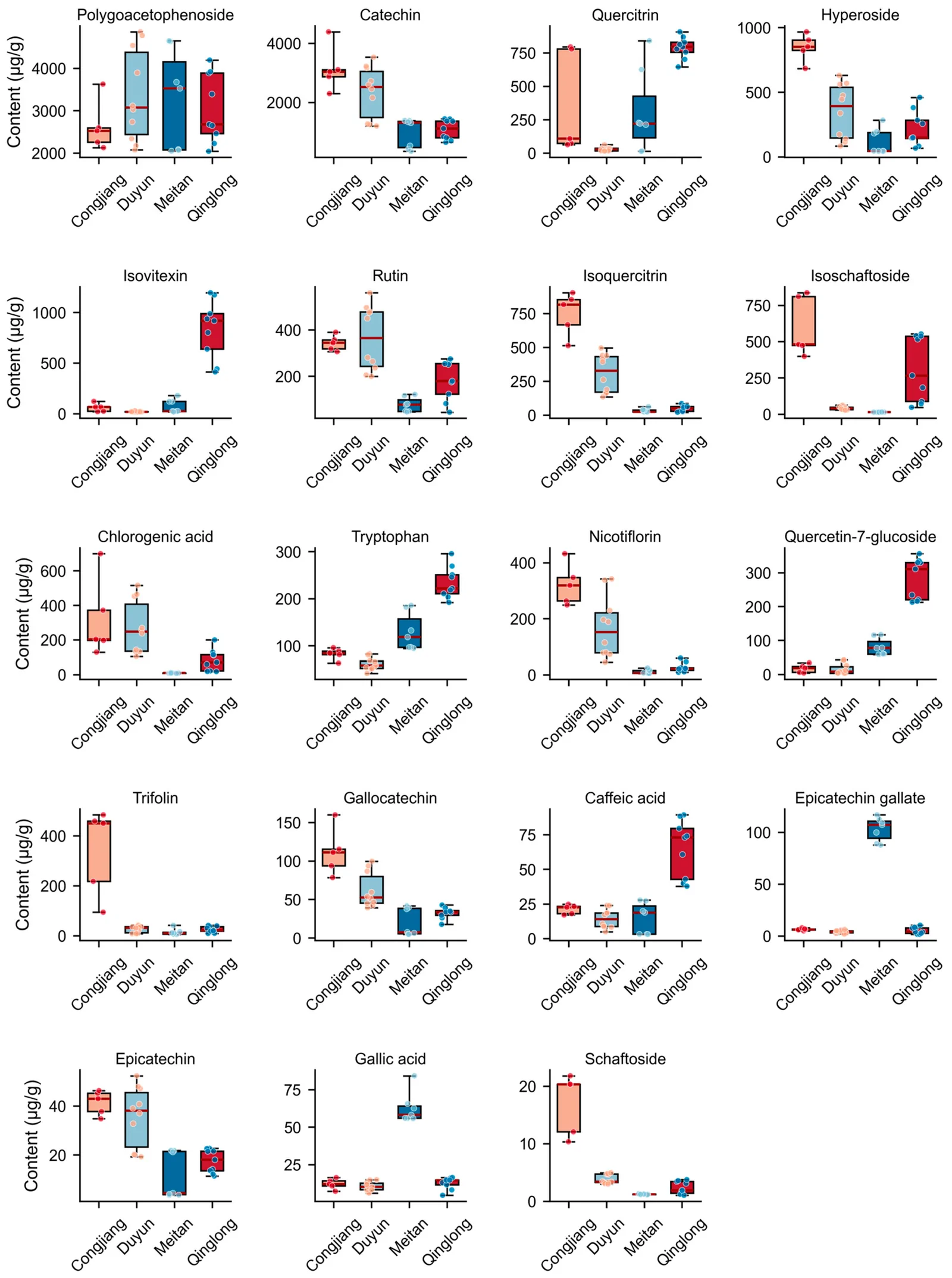
Content of 19 major antioxidants in PML from four regions.

The validated method was applied to quantify the 19 target compounds in 31 batches of PML samples. The chromatogram of the standards is shown in Figure 7. The results showed that all 19 target components were detected across all batches, but the content levels of each component exhibited significant inter-batch differences. The statistical results of the average content for each component in the 31 batches of samples are shown in Table A2 and Figure 8.

In terms of overall content distribution, polygoacetophenoside was the most abundant characteristic component in the samples, with an average content of 3121.23 μg/g across the 31 batches. It was consistently detected in all batches and served as the dominant component in the samples. Among them, sample S9 from Duyun had the highest content, reaching 4856.79 μg/g, while sample S1 from Qinglong had the lowest content at 2041.87 μg/g. This indicates large quality differences in PML from these regions, which may be related to the micro-environment (light, humidity, soil conditions, etc.) at the collection sites. Flavonoids constitute the primary group of bioactive compounds in the samples. Catechin, quercitrin, and hyperoside were the most abundant flavonoids, exhibiting average concentrations of 1798.10 μg/g, 366.79 μg/g, and 340.69 μg/g, respectively.

The highest quercitrin content was found in the S29 sample from Qinglong, reaching 907.94 μg/g; moreover, samples from Meitan had higher average contents of epicatechin gallate and gallic acid than samples from the other three regions. Hyperoside was most abundant in sample S4 from Congjiang, at 966.86 μg/g. In addition, components such as isovitexin, rutin, isoquercitrin, isoschaftoside, chlorogenic acid, tryptophan, nicotiflorin, and quercetin-7-glucoside were also detected at relatively high levels in the samples, with average concentrations all exceeding 90 μg/g. Among them, isovitexin reached 1194.85 μg/g in sample S27 from Qinglong, far exceeding other batches, suggesting significant differences in the accumulation of this component among different batches. Other components, such as trifolin, gallocatechin, caffeic acid, epicatechin, gallic acid, and schaftoside, were consistently detected in the samples, but their overall concentrations were low, with average levels below 80 μg/g. Significant variations in the content of characteristic constituents were observed among different geographic origins, which are likely closely associated with local light intensity, humidity, and soil conditions.

In summary, the composition of phenolic acids, flavonoids, and stilbenoid glycosides in the 31 batches of samples was generally consistent, but there were significant batch-to-batch differences in the content of each component. Among them, polygoacetophenoside was the characteristic high-content component in the samples, and flavonol glycosides and flavanols were the main flavonoid active substances. The results of this study provide comprehensive quantitative data to support subsequent sample quality grade evaluation, active substance screening, and pharmacodynamic material basis research.

## 4. Conclusions

In this study, a 2D-LC-ECD coupled with an UHPLC-Orbitrap-MS/MS platform was used as a robust activity-guided strategy to systematically elucidate the antioxidant constituents of PML and their geographical variation characteristics. The primary constituents with antioxidant activity of PML were identified. In vitro free radical scavenging assays demonstrated that the antioxidant activity of PML was predominantly attributed to its phenolic acids and flavonoids. Notably, 10 key biomarkers were identified using multivariate statistical analysis. Quantitative analysis revealed that geographical origin significantly affected the accumulation of these phytochemicals. Collectively, these findings not only clarify the material basis underlying the antioxidant activity of PML but also establish the identified geographical markers as a solid scientific foundation for quality control, traceability, and the rational development of PML and other medicinal plants.

## Figures and Tables

**Table 1:** Collection sites for the 31 batches of PML samples.

Sample	Collection Location	Sample	Collection Location
S1	Qinglong, Guizhou	S17	Duyun, Guizhou
S2	Qinglong, Guizhou	S18	Meitan, Guizhou
S3	Congjiang, Guizhou	S19	Meitan, Guizhou
S4	Congjiang, Guizhou	S20	Meitan, Guizhou
S5	Congjiang, Guizhou	S21	Meitan, Guizhou
S6	Congjiang, Guizhou	S22	Meitan, Guizhou
S7	Congjiang, Guizhou	S23	Meitan, Guizhou
S8	Duyun, Guizhou	S24	Meitan, Guizhou
S9	Duyun, Guizhou	S25	Qinglong, Guizhou
S10	Duyun, Guizhou	S26	Qinglong, Guizhou
S11	Duyun, Guizhou	S27	Qinglong, Guizhou
S12	Duyun, Guizhou	S28	Qinglong, Guizhou
S13	Duyun, Guizhou	S29	Qinglong, Guizhou
S14	Duyun, Guizhou	S30	Qinglong, Guizhou
S15	Duyun, Guizhou	S31	Qinglong, Guizhou
S16	Duyun, Guizhou		

**Table 2:** Second-dimension liquid chromatography elution gradient.

^1^D Fraction Time Interval (min)	0–6 min (A)	6–7.5 min (A)	7.5–8 min (A)	8–9 min (A)	9–12 min (A)
3~20.5 min of ^1^D	5–20%	20–90%	90%	90–5%	5%
21~33 min of ^1^D	10–25%	25–90%	90%	90–10%	10%
33.5~45 min of ^1^D	15–30%	30–90%	90%	90–15%	15%
45~53 min of ^1^D	20–35%	35–90%	90%	90–20%	20%

**Table 3:** Identification of compounds in PML using UHPLC-Orbitrap-MS/MS.

Peak No.	^1^D Time (min)	MS (*m*/*z*)	Molecular Formula	ppm	MS/MS Fragmentation (*m*/*z*)	Compound Name	Confidence Level
1	3.0–3.5	169.0142	C_7_H_6_O_5_	−0.24	125.0244, 81.0344, 79.0187	Gallic acid	Level I
2	5.0–5.5	305.0667	C_15_H_14_O_7_	0.1	111.0452, 125.0244, 137.0246, 219.0666, 261.0771	Gallocatechin	Level I
3	5.5–6.0	331.0669	C_13_H_16_O_10_	−0.48	169.0142,125.0244, 271.0509	Galloyl glucoside	Level II
4	6.0–6.5	577.1349	C_30_H_26_O_12_	−0.42	125.0244, 289.0715, 407.0767, 451.1032	Procyanidin B	Level II
5	6.5–7.5	341.0087	C_15_H_18_O_9_	1.17	179.0570, 161.0246, 147.9214, 103.9316	1-Caffeoyl-β-*D*-Glucose	Level II
6	8.0–8.5	179.0349	C_9_H_8_O_4_	−0.45	135.0452, 117.0340, 161.0243,	Caffeic acid	Level I
7	8.5–9.0	203.0824	C_11_H_12_N_2_O_2_	−0.98	159.0452, 116.0506, 142.0659	Tryptophan	Level I
8	8.0–11.0	289.0716	C_15_H_14_O_6_	−0.55	245.0820, 271.0627, 203.0714, 109.0295, 137.0244	Catechin	Level I
9	10.5–13.0	353.0875	C_16_H_18_O_9_	−0.85	191.0560, 179.0348, 135.0455	Chlorogenic acid	Level I
10	13.5–14.5	507.1351	C_20_H_28_O_15_	−0.79	345.0824, 331.0383, 315.0150, 183.0297	Polygoacetophenoside glucoside	Level II
11	14.0–14.5	355.1032	C_16_H_20_O_9_	−0.7	193.0505, 178.0271, 149.0609	Ferulic acid glucoside	Level II
12	16.0–17.0	593.1297	C_30_H_26_O_13_	−0.62	289.0716, 245.0817, 305.0667, 407.0767	Epigallocatechin-epicatechin	Level II
13	16.0–17.5	289.0718	C_15_H_14_O_6_	0.14	245.0820, 271.0627, 203.0714, 109.0295, 137.0244	Epicatechin	Level I
14	18.0–18.5	507.1351	C_20_H_28_O_15_	−0.79	183.0297, 350.9373, 129.3007	Polygoacetophenoside glucoside isomer1	Level II
15	21.0–22.5	593.1297	C_30_H_26_O_13_	−0.62	289.0716, 245.0817, 305.0667, 407.0767	Epigallocatechin-epicatechin isomer	Level II
16	23.0–25.5	345.0825	C_14_H_18_O_10_	−0.46	183.0221, 327.0764, 165.0196,	Polygoacetophenoside	Level II
17	24.5–25.5	579.1351	C_26_H_28_O_15_	−0.8	399.0718, 459.0929, 339.0511, 369.0612, 561.1226	Pentahydroxystilbene diglucoside	Level II
18	23.5–25.5	441.0826	C_22_H_18_O_10_	−0.36	289.0727, 169.0150, 125.0247	Epicatechin gallate	Level I
19	26.0–26.5	507.1351	C_20_H_28_O_15_	−0.79	183.0297, 345.0822, 129.3007	Polygoacetophenoside glucoside isomer	Level II
20	29.0–29.5	563.1399	C_26_H_28_O_14_	−1.24	383.0768, 353.0663, 443.0988, 545.1288, 503.1182	Acylated stilbene glycoside	Level II
21	29.5–30.0	579.1353	C_26_H_28_O_15_	0.52	369.0613, 399.0714, 133.0293, 459.0874, 285.0405	Carlinoside	Level II
22	31.5–32.0	563.1404	C_26_H_28_O_14_	0.42	353.0663, 383.0768, 297.0764, 325.0719, 283.0616, 473.1089, 503.1182, 191.0349	Isoschaftoside	Level I
23	33.0–33.5	431.0979	C_21_H_20_O_10_	−0.23	269.0456, 117.0347, 151.0031	Apigenin-O-glucoside	Level II
24	34.5–35.5	497.0933	C_21_H_22_O_14_	−0.8	345.0812, 169.5903, 271.0711	Methyl 4,6-di-O-galloyl-β-*D*-glucopyranoside	Level II
25	36.5–37.0	563.1404	C_26_H_28_O_14_	−0.36	545.1281, 473.1089, 443.0982, 353.0664, 383.0767	Schaftoside	Level I
26	37.0 min	431.0979	C_21_H_20_O_10_	−1.16	413.0877, 341.0667, 311.0561	Isovitexin	Level I
27	39.0–39.5	755.1823	C_36_H_36_O_18_	−0.66	593.1506, 575.1401, 473.1081, 431.0776, 269.0460	Caffeoyl apigenin-7,4′-di-O-β-*D*-glucoside	Level II
28	38.5–39.5	463.0879	C_21_H_20_O_12_	−0.43	301.0346, 300.0376, 179.0086, 151.0037	Hyperoside	Level I
29	39.5–40.0	463.0879	C_21_H_20_O_12_	−0.43	300.0376, 271.0249, 301.0345	Isoquercitrin	Level I
30	39.5–40.0	609.1453	C_27_H_30_O_16_	−1.31	301.0343, 151.0037, 179.0037	Rutin	Level I
31	39.5–40.0	609.1454	C_27_H_30_O_16_	−1.15	463.0488, 301.0344, 179.0042	Quercetin-3-O-neohesperidin	Level II
32	42.0–42.5	463.0877	C_21_H_20_O_12_	−1.08	301.0351, 151.0035, 179.0086	Quercetin-7-glucoside	Level I
33	41.0–42.5	725.1718	C_35_H_34_O_17_	−0.69	563.1404, 425.0878, 179.0350	Caffeoylapigenin-O-glucose-O-arabinoside	Level II
34	41.5–42.0	507.1138	C_23_H_24_O_13_	−1.18	345.0824, 329.0661, 301.0712, 317.0661	5,7,3′,6′-tetrahydroxy-8,2′-dimethoxyflavone-6′-O-glucoside	Level II
35	44.0–44.5	481.0985	C_21_H_22_O_13_	−0.42	305.7862, 153.0198, 185.0343	Gallocatechin-O-β-*D*-glucuronide	Level II
36	44.5–45.0	447.0925	C_21_H_20_O_11_	−0.51	285.0328, 227.0351, 151.0042, 179.2851	Kaempferol glucoside	Level II
37	45.0–45.5	447.0927	C_21_H_20_O_11_	−1.34	301.0349, 151.0036, 179.0056	Quercitrin	Level I
38	46.0–46.5	593.1508	C_27_H_30_O_15_	−0.67	285.0401, 447.0946, 270.8632	Nicotiflorin	Level I
39	46.0–46.5	725.1722	C_35_H_34_O_17_	−0.17	577.1393, 407.0758, 289.0720, 125.0246	Procyanidin dimer monoglucoside	Level II
40	46.5–47.0	625.1193	C_30_H_26_O_15_	−0.96	463.0880, 301.0348, 300.0273, 151.0036	Quercetin-3-O-(6″-O-caffeoyl)-β-*D*-glucoside	Level II
41	48.5–49.0	447.0931	C_21_H_20_O_11_	−0.45	151.0036, 269.0401, 285.0405	Trifolin	Level I
42	48.0–48.5	521.1304	C_24_H_26_O_13_	0.77	327.0720, 193.0505, 183.0298	Feruloyl-galloyl glucose	Level II
43	51.5–52.0	609.1461	C_27_H_30_O_16_	0	447.0940, 285.0401, 151.0038	Kaempferol di-glucoside	Level II

Note: Level I: Structure confirmed by reference standard; Level II: Tentative identification via database matching or SIRIUS CSI: FingerID.

## Data Availability

The original contributions presented in this study are included in the article. Further inquiries can be directed to the corresponding author.

## Appendix A

**Table A1:** Linear equations for 19 major antioxidants in PML.

	Compound	Linearity Range (μg/mL)	Linear Equation	R^2^	LOD (ng/mL)	LOQ (ng/mL)	Precision		Recovery		Matrix Effect (%)
							Intra-Day (%)	Inter-Day (%)	Average (%)	RSD (%)	
1	polygoacetophenoside	0.625–31.25	y = 115,183,703.9x − 17,418,644.3	0.9943	0.06	0.20	1.45	2.21	105.29	2.15	105.47
2	Catechin	0.5–50.00	y = 24,791,449.7x − 3,446,627.4	0.9980	0.4	1.33	0.95	1.69	96.74	0.20	104.42
3	Quercitrin	0.05–10.00	y = 202,157,159.5x + 1,541,227.2	0.9982	0.03	0.11	0.87	1.54	98.8	0.21	107.67
4	Hyperoside	0.125–12.50	y = 21,892,124.4x + 1,568,055.2	0.9983	0.05	0.18	1.19	1.92	93.04	2.65	99.25
5	Isovitexin	0.0625–12.50	y = 42,333,294.4x−1,838,583.7	0.9984	0.02	0.44	1.08	1.79	94.53	0.11	104.33
6	Rutin	0.0625–6.25	y = 13,051,712.4x + 739,040.4	0.9997	0.06	0.20	1.02	1.68	98.17	1.84	102.10
7	Isoquercitrin	0.625–6.25	y = 44,872,558.5x + 317,777.1	0.9989	0.05	0.17	1.41	2.23	94.19	0.20	99.25
8	Isoschaftoside	0.025–6.25	y = 6,973,823.2x + 206,630.9	0.9999	0.11	0.36	1.11	1.84	96.77	0.27	92.05
9	Chlorogenic acid	0.025–5.00	y = 19,973,127.1x + 484,000.5	0.9991	0.36	1.19	1.16	1.88	99.85	0.34	97.67
10	Tryptophan	0.0625–6.25	y = 9,727,897.4x + 379,540.2	0.9969	0.09	0.31	0.91	1.66	96.6	0.22	109.98
11	Nicotiflorin	0.0125–2.50	y = 15,726,276.9x + 37,549.5	0.9991	0.04	0.14	1.03	1.77	97.48	0.97	102.92
12	Quercetin-7-glucoside	0.0125–2.5	y = 14,796,922.0x + 184,670.2	0.9992	0.57	1.90	0.96	1.74	94.58	0.04	99.26
13	Trifolin	0.0125–2.5	y = 22,377,110.5x + 193,810.4	0.9973	0.26	0.88	1.27	2.02	95.58	4.11	107.39
14	Gallocatechin	0.0125–2.5	y = 167,691,074.2x − 1,376,367.6	0.9929	0.08	0.26	0.94	1.52	99.26	1.40	99.98
15	Caffeic acid	0.00625–1.25	y = 34,841,781.4x + 85,213.2	0.9999	0.01	0.03	0.88	1.41	95.46	0.89	106.11
16	Epicatechin gallate	0.0125–2.5	y = 23,138,025.2x + 202,297.4	0.9998	0.08	0.25	1.12	1.86	98.4	0.52	98.99
17	Epicatechin	0.00625–0.625	y = 2,787,207.1x + 13,587.9	0.9982	0.4	1.35	1.18	1.97	102.04	2.50	102.01
18	Gallic acid	0.0125–2.5	y = 22,039,149.0x + 235,673.0	0.9998	0.03	0.1	0.97	1.73	95.88	1.16	100.70
19	Schaftoside	0.0025–0.625	y = 13,692,665.8x + 20,757.6	0.9988	0.08	0.22	0.91	1.66	96.6	0.22	105.47

**Table A2:** Specific concentrations (μg/g) of 19 major antioxidants in 31 batches of PML samples.

Sample	Polygoacetophenoside	Catechin	Quercitrin	Hyperoside	Isovitexin	Rutin	Isoquercitrin	Isoschaftoside	Chlorogenic Acid	Tryptophan	Nicotiflorin	Quercetin-7-glucoside	Trifolin	Gallocatechin	Caffeic Acid	Epicatechin Gallate	Epicatechin	Gallic Acid	Schaftoside
S1	2041.87	649.84	795.30	82.52	637.87	44.65	51.26	552.77	199.85	203.82	46.33	220.29	39.45	33.41	39.85	1.61	11.38	13.00	1.79
S2	2460.70	844.95	779.37	459.75	918.13	82.26	84.80	182.32	70.79	250.92	59.43	329.60	36.13	35.56	42.88	1.52	14.16	4.58	3.10
S3	2127.32	2299.27	795.30	683.69	20.28	305.88	667.74	398.28	129.10	63.48	263.32	6.09	93.92	78.44	17.04	4.87	34.79	7.25	20.33
S4	2595.29	2874.61	779.37	966.86	121.86	389.98	903.24	836.57	371.51	88.98	432.21	34.00	483.86	111.05	23.23	6.23	42.98	14.18	12.07
S5	3624.35	3035.19	72.82	851.80	66.12	356.96	512.54	810.23	699.12	81.16	248.96	4.31	217.56	93.87	24.79	5.90	45.25	16.37	10.34
S6	2523.29	4390.84	62.96	902.76	65.56	344.47	853.46	480.79	203.52	84.53	346.89	23.47	449.84	159.81	18.04	7.85	37.76	12.11	21.80
S7	2259.92	3108.81	108.47	823.20	24.92	318.27	815.92	473.55	196.91	95.89	319.01	18.53	458.94	115.24	22.64	6.74	46.29	10.87	20.33
S8	4536.22	2158.57	62.20	569.17	14.56	479.06	439.82	49.13	452.73	68.86	195.93	3.72	35.74	86.58	17.20	3.51	32.79	13.12	3.26
S9	4856.79	2456.70	19.72	629.72	17.70	560.20	495.38	61.58	516.05	83.02	229.21	3.05	41.93	99.58	18.67	3.95	37.04	14.71	3.43
S10	2341.04	1252.50	28.82	473.95	18.04	474.86	412.75	28.70	257.20	59.23	341.91	8.75	36.22	42.12	24.00	2.89	19.93	6.56	3.26
S11	2173.63	1204.10	34.13	446.05	17.87	449.86	393.58	36.49	238.39	54.19	337.58	7.87	35.53	39.20	23.81	2.53	19.24	6.05	4.73
S12	4768.94	2719.79	37.17	560.78	19.59	496.36	439.72	50.40	463.30	80.72	188.65	3.81	33.20	93.64	18.41	4.34	40.76	14.90	3.36
S13	3030.21	1268.63	36.41	337.64	20.76	280.51	261.13	43.18	268.42	41.77	114.45	4.06	20.60	51.56	10.65	1.84	20.17	8.37	3.05
S14	3892.14	3168.21	28.06	173.02	16.80	205.29	164.48	42.23	133.09	50.90	44.95	26.52	7.70	46.55	4.89	4.21	47.12	10.12	2.98
S15	2735.70	3222.31	17.45	114.21	19.75	236.01	161.62	30.91	132.36	57.40	77.76	41.24	8.37	53.68	8.60	5.55	47.89	10.29	4.66
S16	3112.26	3533.74	17.07	137.88	29.72	263.70	188.03	33.52	142.37	66.14	79.53	12.18	30.53	59.44	9.02	6.22	52.33	11.71	4.81
S17	2075.52	2595.95	15.17	83.79	19.96	199.52	134.11	27.70	104.22	52.14	72.72	42.63	7.93	44.83	8.23	4.98	39.02	8.26	4.95
S18	2086.63	1383.86	17.07	42.96	22.53	47.75	24.17	13.75	12.62	94.27	6.87	59.34	6.34	39.60	3.13	107.21	21.80	55.90	1.21
S19	2066.58	1386.48	13.27	44.73	23.85	48.98	23.66	13.71	9.96	96.92	5.95	57.90	5.97	41.61	3.13	112.36	21.83	58.14	1.13
S20	2047.47	1309.76	212.76	43.90	24.71	46.04	23.18	12.99	7.55	96.47	5.75	60.23	5.26	37.71	3.04	99.80	21.19	55.97	1.11
S21	4627.81	1340.47	226.42	45.35	179.72	116.23	22.55	12.99	8.76	181.89	5.06	116.82	41.25	7.38	27.23	109.11	4.43	55.78	1.20
S22	3524.05	344.93	221.11	182.43	120.72	77.25	36.40	13.56	7.02	118.40	15.71	77.15	10.25	4.81	18.77	87.75	3.62	62.63	1.30
S23	3670.26	417.93	841.19	195.15	121.53	80.63	39.56	13.29	8.20	132.49	16.21	77.78	11.30	4.66	19.82	88.97	3.52	65.57	1.25
S24	4644.67	531.39	625.39	283.55	18.16	121.49	61.01	14.55	9.83	185.67	24.38	115.46	16.02	6.33	27.79	116.81	3.97	84.23	1.20
S25	2674.89	1446.81	645.49	283.53	443.36	174.69	23.16	46.73	17.54	295.60	8.93	335.04	9.49	26.03	60.71	7.76	22.68	14.61	1.40
S26	3389.75	1118.23	870.01	380.70	1173.82	258.45	28.50	87.46	21.12	192.05	21.48	356.18	41.27	42.71	89.29	3.84	18.03	16.15	1.20
S27	2647.79	805.46	701.62	256.48	1194.85	179.04	20.75	71.14	18.37	245.89	16.45	234.04	29.90	29.59	88.29	2.65	13.59	11.58	1.03
S28	4191.87	1440.38	755.86	143.84	988.53	274.80	68.95	539.13	128.87	221.81	25.09	212.81	21.58	38.95	79.53	10.35	22.60	16.44	3.76
S29	2226.47	703.95	907.94	67.27	413.01	123.17	28.62	265.84	59.66	269.24	10.76	310.99	9.00	17.67	37.81	3.97	12.15	8.19	1.89
S30	3886.02	1359.83	829.81	145.88	801.68	250.65	56.55	516.29	111.25	210.70	21.00	215.83	18.51	33.69	73.00	7.48	21.45	14.71	3.43
S31	3918.68	1367.63	812.75	148.89	937.10	254.63	62.67	537.56	115.03	218.32	23.11	329.92	19.88	34.82	74.04	8.29	21.56	14.68	3.57
Mean	3121.23	1798.10	366.79	340.69	274.61	243.28	241.91	203.14	164.99	130.41	116.31	108.05	73.66	51.94	30.24	27.13	25.85	23.13	4.93

**Table A3:** Sample collection site information (altitude, annual average temperature, and coordinates).

Collection Location	Altitude (m)	Annual Avg. Temp (°C)	Coordinates
Qinglong, Guizhou	1500	14.2	25°50′ N, 105°13′ E
Congjiang, Guizhou	240	18.2	25°45′ N, 108°54′ E
Meitan, Guizhou	770	14.8	27°46′ N, 107°28′ E
Duyun, Guizhou	800	16.0	26°16′ N, 107°31′ E

Notes: Altitude and annual average temperature values are from county meteorological stations.
